# The pathology of co-infection with Usutu virus and *Plasmodium* spp. in naturally infected Eurasian blackbirds (*Turdus merula*)

**DOI:** 10.1016/j.onehlt.2023.100534

**Published:** 2023-04-01

**Authors:** Gianfilippo Agliani, Giuseppe Giglia, Erwin de Bruin, Tjomme van Mastrigt, Rody Blom, Reina S. Sikkema, Marja Kik, Marion P.G. Koopmans, Andrea Gröne, Judith M.A. Van den Brand

**Affiliations:** a Division of Pathology, Department of Biomolecular Health Sciences, Utrecht University, Utrecht, the Netherlands; b Department of Animal Ecology, Netherlands Institute of Ecology (NIOO-KNAW), Wageningen, the Netherlands.; c Vogeltrekstation - Dutch Centre for Avian Migration and Demography (NIOO-KNAW), Wageningen, the Netherlands.; d Wildlife Ecology and Conservation Group, Wageningen University & Research, Wageningen, the Netherlands.; e Laboratory of Entomology, Plant Sciences Group, Wageningen University & Research, Wageningen, the Netherlands; f Department of Viroscience, Erasmus Medical Center, Rotterdam, the Netherlands; g Dutch Wildlife Health Centre, Utrecht University, Utrecht, the Netherlands

**Keywords:** Arboviruses, Zoonosis, Co-infection, Avian malaria, Flaviviruses

## Abstract

Usutu virus (USUV) is a mosquito-borne zoonotic flavivirus causing mortality in Eurasian blackbirds (*Turdus merula*) in Europe. In dead blackbirds, avian malaria co-infection due to mosquito-borne hemosporidians (e.g., *Plasmodium* spp.) has been reported. In humans, a similar co-infection of a flavivirus, Dengue virus, and *Plasmodium* spp. is causing increased severity of clinical disease. Currently, the effects of co-infection of arboviruses and hemosporidians in blackbirds remain unclear. This study investigates the rate of USUV and *Plasmodium* spp. co-infection in found-dead blackbirds (*n* = 203) from 2016 to 2020 in the Netherlands. Presence of *Plasmodium* spp. was evaluated by cytology (43/203; 21,2%), histopathology (94/186; 50,5%) and qPCR (179/203; 88,1%). The severity of histological lesions in USUV and *Plasmodium* spp. co-infected dead blackbirds (121/203; 59,6%) were compared with those in *Plasmodium* spp. single-infected cases. Additionally, since no knowledge is present on the infection rate on live birds and mosquitoes in the Netherlands, a small group of live blackbirds (*n* = 12) and selected in the field-collected mosquito pools (*n* = 96) in 2020 were tested for the presence of *Plasmodium* spp. The latter was detected in the tested live blackbirds by qPCR (8/10; 80%), and cytology (3/11; 27,3%) and in the mosquito pools by qPCR (18/96; 18,7%). For this study, co-infection between USUV and *Plasmodium* spp. was observed only in the dead blackbirds. The high *Plasmodium* spp. presence, associated with lower lesions score, in single infected found dead birds suggest a predominantly smaller pathogenic role as single agent. On the other hand, the higher histological lesion scores observed in USUV and *Plasmodium* spp. co-infected birds suggests a major pathogenic role for the virus or an increased severity of the lesions due to a possible interplay of the two agents.

## 1 Introduction

Usutu virus (USUV) is an emerging zoonotic mosquito-borne arbovirus (Family Flaviviridae, genus *Flavivirus*), that can cause disease and mortality mainly in passerine birds [1] and raptors in Europe [[2], [3], [4], [5], [6], [7]]. USUV was first identified in 1959 in *Culex neavei* mosquitoes in former Swaziland [8]. Its first detection in Europe dates to 2001, when an outbreak of disease with high mortality in passerines was reported in Vienna, Austria [2]. From retrospective studies on archived blackbird tissues, USUV was detected in the Tuscany region, Italy in 1996, suggesting an earlier introduction in Europe [9]. In recent years, continuous circulation has been reported in Europe [10], where its enzootic transmission cycle is sustained by mosquitoes (*Culex* spp.) as vectors and several bird species as amplifying host. In the Netherlands, USUV was first reported in 2016, and is primarily associated with mortality in Eurasian blackbirds (*Turdus merula*) [6,7] and Great Gray Owls (*Strix nebulosa*) [6]. Tissue lesions described for USUV-infected birds were mainly foci of hepatocellular and splenic necrosis, myocardial necrosis and myocarditis, pneumonia and a lymphoplasmacytic encephalitis [5,7,11,12].

In blackbirds, avian malaria has been reported as a highly common co-morbidity [6,7,13]. Avian malaria is a common disease of birds with a variable etiology in which different genera of malaria parasites (hemosporidians) are identified, such as *Plasmodium* spp. which is the main pathogenic agent in birds [14]. Many bird species belonging to various families and orders are considered susceptible [[15], [16], [17]]. Despite the high rate of infection with hemosporidians described in the last years in Europe [7,13], the pathogenic effect of the *Plasmodium* spp. in the host is variable and still not fully understood. Avian malaria associated with tissue lesions and fatal disease has been reported in different avian species [15,[17], [18], [19]], with inflammation and necrosis in various affected organs (e.g., heart, spleen, liver, lung, brain), sometimes in clear association with micro-thrombosis and development of extraerythrocytic tissue stages [[15], [16], [17],[20], [21], [22]]. In birds, both USUV and *Plasmodium* spp. infections show an overlapping pattern of lesions posing a challenge in the diagnosis and possibly in the definition of the actual cause of damage and death.

The transmission of *Plasmodium* spp.*,* as well as USUV, is mediated by mosquitoes (e.g., *Culex* spp.) acting as the main biological vector in Europe [23]. For USUV, data on vector abundance and virus positivity suggest a higher presence of the vector and virus circulation during Summer (July–September) [24,25]. Similarly, *Plasmodium* spp. detection increases during late summer and beginning of autumn (August–October) [26]. The close relationship between both, USUV and *Plasmodium* with their vector, results in a highly similar seasonality in the detection, infection and disease outbreak occurrence.

Flavivirus and *Plasmodium* spp. co-infections in humans have been previously reported mainly in Africa, India and South America [[27], [28], [29]]. Of these, Dengue virus and *P. falciparum* co-infection is one of the most concerning since an increase of clinical disease severity has been described for several human cases [27,29]. The similarities of the lesion patterns observed for the two agents possibly suggest, as in human clinical disease, a summative effect of the co-infection enhancing the lesion severity also for blackbirds, which so far remains unclarified.

This research aims to evaluate the effect of the co-infection comparing the severity of tissue lesions in USUV and *Plasmodium* spp. co-infected dead blackbirds with *Plasmodium* spp. single-infected cases. In addition, it investigates the prevalence of *Plasmodium* spp. and USUV in found dead blackbirds from 2016 to 2020 in the Netherlands and screened the presence of *Plasmodium* spp. infection in a selected subset of wild caught live blackbirds and mosquito pools in 2020.

## 2 Materials and methods

### 2.1 Animals and sampling materials

Between 2016 and 2020, dead blackbirds (*N* = 203) were submitted to the Dutch Wildlife Health Centre (DWHC) to monitor circulating diseases in wild animals in the Netherlands [6,7]. The blackbirds were necropsied, organs were collected and both stored at −80 °C for molecular detection or fixed in 10% neutral buffered formalin for histopathology.

Wild blackbirds, both male and female, were caught using mist nets in 2020 in the Netherlands, sampled and equipped with a uniquely numbered aluminum ring (Dassenbos, Wageningen, the Netherlands: 51°58′59.0”N, 5°39′19.3″ E). The birds were bled by brachial venipuncture (KNAW-DEC licensed under protocol AVD801002015342 to H. van der Jeugd). Directly after bleeding, approximately 5 μL of blood was directly smeared onto a slide for later examination. A dry blood spot (DBS) card was used to sample and store the blood for molecular detection of *Plasmodium* spp. in live birds. A selected subset (*N* = 12) of the birds, based on the time of collection (June–December 2020) and the availability of DBS cards and blood smears was tested for the identification of *Plasmodium* spp. For each individual bird, only one sample was included in the analysis to avoid pseudo-replication.

At 16 bird sampling sites in the Netherlands, mosquitoes were trapped for 15 weeks, from early July 2020 until early October 2020. At each site, mosquitoes were trapped for one night per week, using BG-Pro (Biogents GmbH, Regensburg, Germany) traps, using molasses-fermentation (250 g of molasses, 17.5 g instant dry yeast, and ∼ 1.8 L tap water) as CO_2_-source [30]. Mosquitoes were morphologically identified following the identification key of Becker et al. (2020) [31]. Since adult *Culex pipiens* and *Culex torrentium* females are morphologically nearly identical, they were classified as *Cx. pipiens/torrentium*. Subsequently, mosquitoes were pooled in pools with a maximum of ten mosquitoes per pool. Pools of *Culex pipiens/torrentium* (*N* = 96) were selected from each sampling location based on the time of collection (July–September 2020) when higher prevalence of *Plasmodium* spp. in mosquitoes is expected [26].

### 2.2 Histopathology and cytology

For histopathology, after routine processing and paraffin embedding, 3–5 μm sections of all tissues were routinely stained with haematoxylin & eosin (H&E). To determine the severity of the lesions, a semi-quantitative scoring system was defined based on USUV and *Plasmodium* spp. most affected organs (liver, spleen, heart, brain, lung) as previously reported [7]. Additionally, presence of *Plasmodium* spp. specific features (malarial pigment and extra-erythrocytic stages of the parasite) were identified based on literature criteria [32] and subsequently scored with the same system, in brief: severity was estimated based on percentage of tissue affected as follows: absent (grade 0; normal tissue), mild (grade 1; < 20%), moderate (grade 2; 20–60%) and severe (grade 3; > 60%).

For the cytological direct detection of hemosporidians, impression smears of liver, spleen and lung of the found dead birds were performed during necropsy, and rapidly stained with Hemacolor (Merck, Darmstadt, Germany) according to a standard manufacturer's protocol. Blood smears prepared from blood samples of 12 live blackbirds and stained with the May Grünwald-Giemsa staining according to a standard protocol were available for hemosporidian detection.

### 2.3 USUV and hemosporidians molecular detection and sequencing

#### 2.3.1 Usutu virus

For USUV, molecular detection was performed as described previously by Oude Munnink et al. 2020 [33]. Brain tissue samples (*N* = 200) collected from blackbirds during post-mortem investigation were homogenized in 300uL tissue lysis buffer (MagNA Pure DNA Tissue Lysis Buffer) using the Fastprep bead beater (4.0 m/s for 10 s), RNA extraction and USUV RT-PCRs were performed with an additional dilution step, where 60uL homogenized sample was added to 540uL VTM (or DMEM) and 600uL External lysis buffer. Phocine distemper virus (PDV) was used as an internal control [34,35]. Throat and cloaca swabs of live blackbirds were stored in Virus Transport Medium (VTM). 600 uL of MagNA Pure 96 External Lysis Buffer (Manufactured by Roche: https://lifescience.roche.com/en_nl/products/magna-pure-96-external-lysis-buffer.html) was added to 600 uL of sample and RNA extraction was performed on a MagNA Pure 96 Instrument (MagNA Pure 96 Instrument (roche.com)). USUV RT-PCRs [34,35] were performed, using Phocine distemper virus (PDV) as an internal control. For USUV investigation, mosquito pools were tested according to the protocol reported by Blom et al., 2023 [36].

#### 2.3.2 Hemosporidians

For the molecular detection of *Plasmodium* spp., DNA was extracted from DBS cards (*N* = 11) using a DNeasy blood kit (Qiagen) for live blackbirds, and a DNeasy tissue kit (Qiagen) for samples of lung (*N* = 184) and liver (*N* = 185) collected from dead blackbirds and stored at −20 °C and pools of homogenized mosquitoes (*N* = 96), following the manufacturer's instructions.

A novel real-time qPCR method was used for the detection of *Plasmodium* spp. using the non-coding region of mitochondrial DNA, adapted from Ciloglu et al. (2019) [37]. Two microliter of extracted DNA was added to a PCR mix containing 6 pmol of primer, 4 pmol of probe in iTaq universal probes reaction mix (Biorad, California, USA). Primers used for amplification were: forward primer PMF (5′- TGCAGGACGGAGATTACCCGAC-3′) and reverse primer PMR (5′- ACCCGGGAAACCGGCGCTAC-3′). For confirmation of the product a probe was added, PMP (5′- CCGGCATCAATGATAAACGGCGG-3′), labelled with a 6FAM (5′-end) and blackhole quencher 1 (3′-end). Thermal cycling protocol was performed using a CFX connect (Biorad, California, USA) and started with 95 °C for 5 min, a denaturing at 95 °C for 5 s, annealing at 56 °C for 3 s and extension at 60 °C for 30 s were repeated for 45 cycles. For typing and confirmation of the qPCR results, a nested PCR (reference method) was used to amplify a part of the cytochrome *b* gene [38]. PCR products from the nested-PCR were analyzed on 2% agarose gels to determine positivity (expected band size 525 bp). Products from the nested PCR were sequenced through an external Sanger sequencing service (Macrogen, the Netherlands) on a subset of 4 dead blackbirds to confirm the presence of *Plasmodium* spp. suggested by the histological evidence of *exo*-erythrocytic stages morphologically compatible with this genus of parasites.

### 2.4 Statistical analysis

All statistical analyses were performed using SPSS 28.0 software (IBM, SPSS Inc., Chicago, IL, USA). To compare differences in the lesion severity scores between *Plasmodium* spp. single infected and co-infected animals, a Mann-Whitney *U* test was performed. Blackbirds were considered in the group of *Plasmodium* spp. single infection, when histology and qPCR were positive for *Plasmodium* spp. and negative for USUV. Fischer-Freeman-Halton Exact Test was used to assess association between malarial pigment (hemozoin) and presence of exoerythrocytic stages of *Plasmodium* spp. The statistical significance of each test was set at *p* ≤ .05.

## 3 Results

### 3.1 Histopathology and cytology

Upon histology, exoerythrocytic parasites morphologically compatible with *Plasmodium* spp. were detected in 94/186 (50,53%) examined birds (due to autolysis, tissues were not available for all 203 birds). Of the histologically positive birds, 91/94 (96.8%) were also positive with the qPCR.

**Fig. 1:**
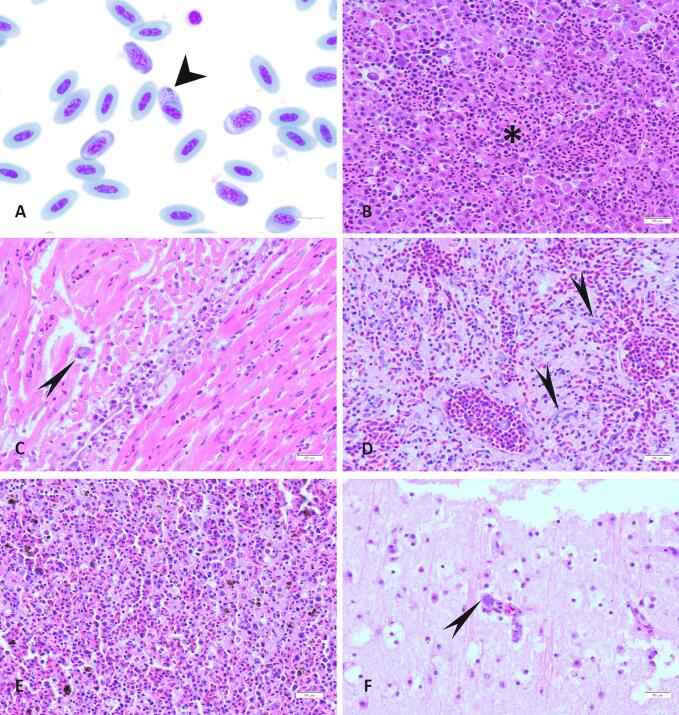
Lesion patterns and parasitic elements in various tissues of blackbirds infected with *Plasmodium* spp. A. Blood smear from a live blackbird; An erythrocyte shows a round (about 4–6 μm in diameter) light bluish intracytoplasmic element (gametocyte) (short arrowhead) associated with malarial pigment (hemozoin) (x1000). B—F. Histological section of: B. liver; Focal-extensive area of necrosis (*) associated with inflammatory infiltrates and exoerythrocytic *Plasmodium* spp. stages (x400) represented as intracytoplasmic oval structure, up to 60 μm in diameter, containing numerous round 1 μm basophilic elements (merozoites). C. myocardium; A single intra-histiocytic exoerythrocytic *Plasmodium* spp. stage (long arrowhead) is seen in areas of myocarditis (x400). D. lung; Numerous exoerythrocytic *Plasmodium* spp. stage (phanerozoite) in pulmonary capillaries (long arrowhead) represented as an elongated structure, up to 80–100 × 20 μm, containing numerous round 1 μm in diameter basophilic elements (merozoites), are associated with pulmonary edema (x400). E. spleen; Multifocal necrosis and aggregates of brown granular malarial pigment (hemozoin) (x40). F. brain; exoerythrocytic *Plasmodium* spp. stage (phanerozoite) (long arrowhead) in a cerebral capillary surrounded by rare lymphocytes (x400).

Exoerythrocytic tissue stages (phanerozoites) were observed in endothelial cells (in lungs and brains) and in macrophages (in liver and spleen) (Fig. 1). Most commonly, exoerythrocytic tissue stages were seen in the lung (83/94; 88,29%) and in the liver (42/94; 44,68%). Additionally, the presence of hemozoin (*Plasmodium*-related pigment) was significantly associated with the presence of the parasite (*p* < .001). Regarding lesion severity, the score was significantly (p < .001) higher in the co-infected birds, for both necrosis and inflammation, in tissue sections of spleen, heart and brain.

On cytological examination of blood smears from live and dead birds, intra-erythrocytic hemosporidian gametocytes, with morphology consistent with *Plasmodium* spp. [39] were seen in 43/203 (21,2%) dead blackbirds and in 3/11 (27,3%) live blackbirds.

### 3.2 USUV and hemosporidian molecular detection and sequencing

**Fig. 2:**
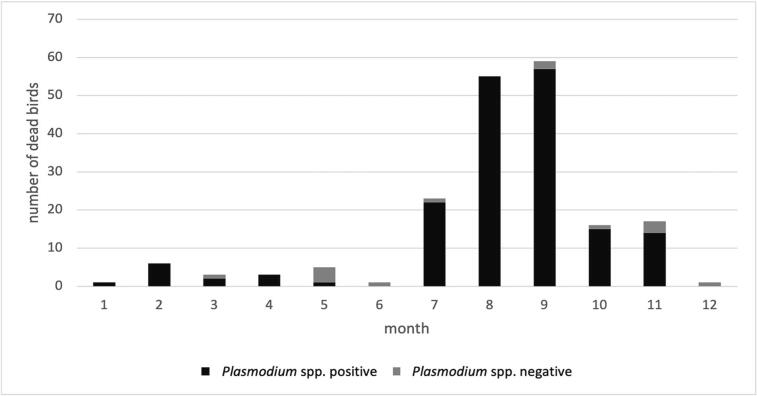
Year-round (2016–2020) presence of *Plasmodium* spp. in e ither the liver or the lung of found dead Eurasian blackbirds.

In the dead blackbirds collected in 2016–2020, 129/203 (63,5%) were positive for USUV [7]. *Plasmodium* spp. was detected in 179/203 (88,1%) of the dead blackbirds in qPCR. Specifically, for the liver 169/203 (83,2%) were positive and similar results were seen for the lung 167/203 (82,2%). The total number of positive animals for both USUV and *Plasmodium* was 121/203 (59,6%), *Plasmodium* spp. single infection was reported in 52/203 (25,6%) blackbirds, no USUV single infected animals were identified in our evaluation and only 14 (6,8%) of the birds were negative for both agents, as reported in Table 1. The dead blackbirds were sent in during all months of the year, however, although *Plasmodium* spp. infections can be detected almost year-round in blackbirds in the Netherlands, a clear peak incidence was observed in late summer to autumn which corresponded to the period of the year when USUV was detected, namely from July to October (Fig. 2) [7]. Based on the results of sequencing of amplified cytochrome B gene (GenBank accession numbers: OQ674234 - OQ674237), all 4 samples examined cluster within the group of avian *Plasmodium* spp.

**Table 1:** USUV and *Plasmodium* spp. co-infection rate in dead Eurasian blackbirds between 2016 and 2020.

		*Plasmodium* spp.	
		Positive	Negative
USUV	Positive	121/203 (59,6%)	0/203 (0%)
	Negative	52/203 (25,6%)	14/203 (6,9%)

To determine the presence of *Plasmodium* spp. in live birds, blood samples from live blackbirds were tested and 8/10 (80%) were positive. 18/96 (18,75%) pools collected from 7 different location, between July and September 2020, with 161 individuals in total were found positive for *Plasmodium* spp. by qPCR. None of the live blackbirds and mosquito pools selected based on our criteria were positive for USUV (unpublished data), therefore evaluation of co-infection in these cases was not possible in the present study. Data regarding infection for *Plasmodium* spp. in live and dead blackbirds and mosquitoes are summarized in Table 2.

**Table 2:** Summary of *Plasmodium* spp. positive live and dead Blackbirds and mosquitoes.

	Live blackbirds		Dead blackbirds			Mosquitoes
	Cytology	qPCR	Cytology	Histology	qPCR	qPCR
*Plasmodium* spp.	3/11 (27,3%)	8/10 (80%)	43/203 (21,2/%)	94/186 (50,5%)	179/203 (88,1%)	18/96 (18,7%)

### 3.3 Statistical analysis

**Fig. 3:**
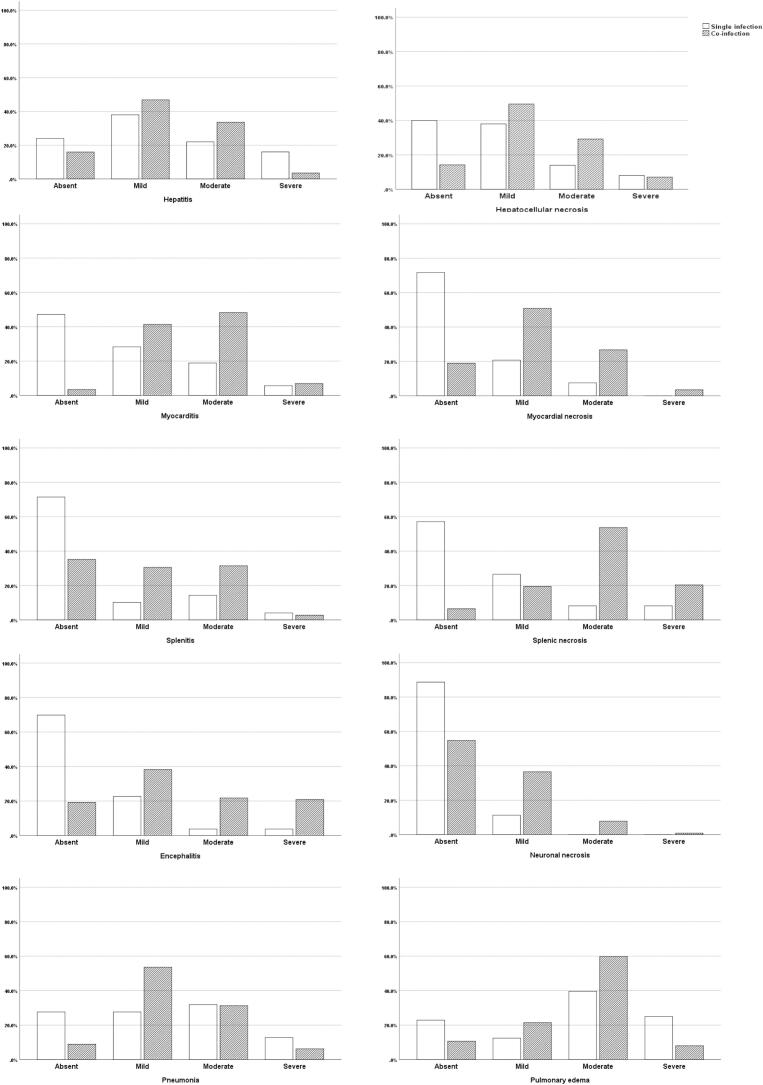
Lesion severity scores by histopathology of *Plasmodium* spp. single infected and USUV co-infected blackbirds.

Results of the statistical analysis showed that the hepatocellular necrosis score was higher in the co-infection group than in the single infection group (*p* < .050). The score of hepatitis was not increased in cases of co-infection (*p* > .050). Myocardial necrosis (*p* < .001), myocarditis (*p* < .001), splenic necrosis (*p* < .001) and splenitis (*p* < .001), as well as encephalitis (*p* < .001) and necrosis (*p* < .001) in the central nervous system were significantly higher in cases of co-infection compared to *Plasmodium* spp. single infection (Fig. 3)

Association was statistically significant for the presence of exoerythrocytic stages and malarial pigment (hemozoin) in the liver, spleen, heart, brain, lungs (p < .001). The presence of exoerythrocytic stages of the parasite and the score of extramedullary haematopoiesis was not significant for any of the organs examined (*p* > .050).

## 4 Discussion

Co-infection with USUV and hemosporidian parasites has been described in blackbirds [13]. This study demonstrates that in the Netherlands a large percentage of found dead blackbirds (2016–2020) was infected with USUV and *Plasmodium* spp. and furthermore highlighted a higher lesions severity in co-infected blackbirds compared to *Plasmodium* spp. single infected ones. Additionally, the presence of *Plasmodium* spp. was evaluated in a selected subset of live caught blackbirds and mosquito pools, sampled in the Netherlands in 2020.

In birds, USUV and *Plasmodium* spp. infection are both associated with similar patterns of damage [7,[15], [16], [17],[20], [21], [22]]. Similar tropism is also seen since both, USUV and *Plasmodium* spp. infect host's endothelial cells and macrophages [7,40]. This study showed an increased severity of lesions in multiple organs (liver, spleen, heart, brain, and lungs) when both USUV and *Plasmodium* spp. were present, compared to cases only infected with *Plasmodium* spp. that showed only mild damage. The reason of the increased severity of lesions in cases of co-infection may be the sum of the damage (necrosis and inflammation) caused by the virus and the parasite. Another possibility would be a possible enhancing effect of one agent on the other (e.g., decrease immune response and increase viral or parasitic replication). As reported in literature, the shared tropism for endothelial cells and macrophages of USUV [7] and *Plasmodium* spp. [40] could suggest in case of co-infection a mechanism of increasing virus uptake in cells, facilitation of entry in tissue cells in the hosts, possibly influencing each other's pathogenicity. Suppression of host T-cell mediated immune response due to *Plasmodium* spp. infection is reported in humans and murine models of malaria [41]. This was not observed in birds experimentally infected with hemosporidians, however, the long-term costs of maintaining an immune reaction during a chronic infection can impair the building of an efficient response against other agents even more so in wild animals with low food resources and higher stress [42].

Due to the absence of cases with single USUV infection in this study, the understanding of the specific single effect of USUV is difficult to compare to the effect of the co-infection with *Plasmodium* spp.; nevertheless, despite the lack of a group of single USUV infection cases, more severe lesions were present in the co-infected cases suggesting a causative relationship with USUV activity (e.g., neuronal necrosis) rather than with *Plasmodium* spp. alone or a possible interplay between the two pathogens, whose nature needs to be further investigated.

A similar pattern as seen in the blackbirds was demonstrated in human co-infection between *Plasmodium* spp. and Dengue virus (DENV) which is associated with an increased severity of clinical disease in co-infected individuals compared to single infected ones [27]. Little is known on flavivirus and *Plasmodium* co-infection associated pathology and on the possible interplay of the two pathogens. Even though *Culex* mosquitoes often feed on humans, the latter do not become infected with avian *Plasmodium* species, nevertheless the study of the pathology and pathogenetic mechanisms behind the co-infections in birds can provide useful insights to better understand the effect of co-infection on the associated human diseases.

To confirm the presence of *Plasmodium* spp. parasites observed in histology, a novel qPCR performed in this study was designed to be specific for *Plasmodium* spp. and highlighted a high parasite prevalence in dead blackbirds in the Netherlands. Next to that, sequencing was performed on selected cases and confirmed *Plasmodium* spp.. Identification of the species of *Plasmodium* involved in the infection represents an important further step of the investigation on co-infections. For instance, some host-adapted species of *Plasmodium* are minimally pathogenic for birds [43], while others have been described as pathogenic for wild birds in both natural and experimental infection. *Plasmodium relictum* has been associated with decline of birds' population in both European [44] and non-European [45] countries, and high mortality was observed in wild birds experimentally infected with *Plasmodium homocircumflexum* [46]. Furthermore, multispecies *Plasmodium* infection in the same bird are reported in literature [47] and might influence the severity of the associated disease. The investigation of the role of specific *Plasmodium* lineages in in both co-infected and single infected birds goes beyond the scope of this paper, nevertheless, remains an important open question for future investigations.

Due to the high variability in lineages, the pathogenic potential of *Plasmodium* spp. in nature is not fully established. Therefore, *Plasmodium* spp. was tested in the blood of live blackbirds caught in a project during a selected time period to compare with the dead birds in the same period (2020). A similar rate of positive samples was identified by cytology and qPCR between live and dead blackbirds, with higher sensitivity of the qPCR. The similar rate of *Plasmodium* spp. detection in dead blackbirds' liver and lungs suggest their comparable suitability for diagnostic purposes. Co-infection between USUV and *Plasmodium* spp. was not observed in the live blackbirds included in the present study, however the high number of live animals infected with *Plasmodium* spp. suggests a low pathogenic effect of the parasite in blackbirds, nevertheless, the parasite can act as population modulator reducing the fitness and increasing the susceptibility to other pathogens of infected individuals [42]. Only few studies investigated co-infection between flaviviruses such as WNV and USUV and *Plasmodium* spp. and the mutual effect of the two agents remains unclear [6,48,49]. Several studies assessed the prevalence of blood parasites and their impact on wild bird populations in Europe [44,50]. One study reported higher prevalence of hemosporidians in live blackbirds through PCR-RFLP [51] compared to blood smears in concordance to this study suggesting a higher sensitivity of the PCR. Limitations of the direct identification of hemosporidians (e.g., *Plasmodium* spp.) on microscopy are due to the limited amount of tissue examined, reducing the chances of detection and appointing the PCR as elective choice for parasite screening in birds. Nevertheless, when PCR is not available, a combination of cytological and histologic examination could increase the chance of *Plasmodium* spp. detection.

Several mosquito species are reported as possible vectors of *Plasmodium* spp. while in Europe [52] a higher prevalence is seen in *Cx. pipiens* mosquitoes compared to others [48,50]. According to mosquitoes' host preference studies, *Cx. pipiens* in Europe feed mainly on birds, including blackbirds as one of the most reported species [53,54]. In the selected mosquito pools sampled within the same period as the live birds in 2020 in the Netherlands, a high presence of *Plasmodium* spp. (18,7%) in *Cx. pipiens* and *Cx. torrentium* mosquitoes was detected similarly to what was reported in other European countries [26,50,55]. Since a strict seasonality is reported for *Plasmodium* spp. prevalence in the vector, for this study, were selected mosquitoes sampled between late Summer and beginning of Autumn, the period during which higher prevalence of the parasite is reported in the vector [26]. Furthermore, high prevalence of the parasite in the vector correlates with the high prevalence in blackbirds observed in the present study. Identification of co-infection between USUV and *Plasmodium* spp. was not applicable to the present study due to lack of positive USUV mosquito pools and remains an interesting question for future investigations.

## 5 Conclusion

Co-infection of USUV and *Plasmodium* spp. caused more severe lesions compared to *Plasmodium* spp. single infection suggesting either a possible summative pathogenic effect or an interplay of the two pathogens. From a One-Health perspective, this study suggests similarities in the more severe disease of co-infection between flaviviruses and *Plasmodium* spp. as was seen in blackbirds with USUV and in humans with DENV. This may be important in future outbreaks with such pathogens in the light of climate change and the spread of mosquito borne diseases. For pandemic preparedness, next to investigating dead birds for monitoring and surveillance purposes, investigating live birds and mosquitos for mosquito borne pathogens, including flaviviruses but also *Plasmodium* spp. is needed.

## Author statement

GA and GG conceived the idea of the manuscript, conceptualization, formal analysis, writing and review of the manuscript. EB, RS, TM and MK contributed with methodology, investigation, and review of the manuscript. MPGK, AG and JvdB contributed with conceptualization, funding acquisition, and review of the manuscript.

## Funding

This publication is part of the project ‘Preparing for vector-borne virus outbreaks in a changing world: a One Health Approach’ (NWA.1160.1S.210) which is (partly) financed by the Dutch Research Council (NWO).

## Declaration of competing interest

The authors declare that they have no known competing financial interests or personal relationships that could have appeared to influence the work reported in this paper.

## Data Availability

Data will be made available on request.
